# Estimating Nurse Workload Using a Predictive Model From Routine Hospital Data: Algorithm Development and Validation

**DOI:** 10.2196/71666

**Published:** 2025-07-31

**Authors:** Paul Meredith, Christina Saville, Chiara Dall’Ora, Tom Weeks, Sue Wierzbicki, Peter Griffiths

**Affiliations:** 1 School of Health Sciences, Faculty of Environmental and Life Sciences, University of Southampton, Building 67, University Road, Southampton, S017 1BJ, United Kingdom, 44 23 8059 5903, 44 23 8059 8909; 2 Workforce & Health Systems, National Institute for Health Research Applied Research Collaboration (Wessex), Southampton, United Kingdom; 3 Portsmouth Hospitals NHS Trust, Portsmouth, United Kingdom; 4 Isle of Wight NHS Trust, Newport, United Kingdom

**Keywords:** workload, staffing, nursing staff, safer nursing care tool, predictive model

## Abstract

**Background:**

Managing nurse staffing is complex due to fluctuating demand based on ward occupancy, patient acuity, and dependency. Monitoring staffing adequacy in real time has the potential to inform safe and efficient deployment of staff. Patient classification systems (PCSs) are being used for per shift workload measurement, but they add a frequent administrative task for ward nursing staff.

**Objective:**

The objective of this study is to explore whether an algorithm could estimate ward workload using existing routinely recorded data.

**Methods:**

Anonymized admission records and assessments from a PCS supporting the safer nursing care tool were used to determine nursing care demand in medical and surgical wards in a single UK hospital between February 2017 and February 2020. Records were linked by ward and time. The data were split into a training set (75%) and a test set (25%). We built a predictive model of ward workload (as measured by the PCS) using routinely recorded administrative data and admission National Early Warning Score. The outcome variable was ward workload derived from the patient classifications, measured as the number of whole-time equivalent (WTE) nursing staff per patient.

**Results:**

In a test set of 11,592 ward assessments from 42 wards with a mean WTE per patient of 1.64, the model’s mean absolute error was 0.078, with a mean percentage error of 4.9%. A Bland-Altman plot of the differences between the predicted values and the assessment values showed 95% of them within 0.21 WTE per patient.

**Conclusions:**

Predictions of nursing workload from a relatively small number of routinely collected variables showed moderate accuracy for general wards in 1 English hospital. This demonstrates the potential for automating assessments of nurse staffing requirements from routine data, reducing time spent on this nonclinical overhead, and improving monitoring of real-time staffing pressures.

## Introduction

Nursing workload management is complex and involves the processes of forecasting, scheduling, staffing, and monitoring [1]. Monitoring a ward’s nursing workload in real time or near time is difficult because of patient movements and because of changes in patient acuity and care needs. This is important because there is a body of evidence that shows nurse understaffing is associated with adverse outcomes for patients and for staff [2,3]. Monitoring supports the identification of staffing deficiencies and informs decisions concerning the deployment or redeployment of resources to address them.

Common structured methods of monitoring staffing requirements make use of patient classification systems (PCSs). Through these systems, care intensity is determined for each patient and is then summed to calculate the total care demand for the ward. Examples are the acuity or dependency assessments in the safer nursing care tool (SNCT) [4], the Oulu Patient Classification qualisan (OPCq) used in RAFAELA [5] and the Perroca Patient Classification System [6]. Assessing and documenting the nursing intensity for every patient on the ward is an additional task for ward nurses, often repeated every shift. Automating the nursing intensity and workload monitoring using data already recorded has the potential to free up valuable staff time in the provision of patient care.

There is little published work on automating PCSs used to measure nursing intensity and workload. However, a model was developed to determine each patient’s workload category as defined by the Perroca Patient Classification Instrument (PPCI) from routine electronic health record data in a Brazilian hospital [7]. A random forest classification model used electronic variables chosen as indicators for each of the 9 care areas in the tool. The classifier was 72% accurate when categorizing the patients overall with a c-statistic of 0.82. An individual patient misclassification may not have much effect on the ward workload estimation as a whole, but there were no details of how to translate the patient workload categories into nurse staffing requirements for wards.

Our aim was to build a predictive model using routine electronic data from 1 hospital, which might be known in real time to estimate nurse staffing requirements for wards. Our objective was to match the recorded values determined by using the patient classification instrument from the SNCT, the most widely used tool in England for determining staffing requirements based on patient need [8]. The SNCT originated as a way of estimating nurse staffing establishment requirements (whole-time equivalent (WTE) posts) by means of twice-a-year audits of ward workload. It included a PCS, which is now in use once, twice, or thrice a day in many hospitals in England to monitor ward workload.

## Methods

### Ethical Considerations

This research was a secondary analysis of data collected for a study that considered the costs and consequences of different nursing staff configurations (Trial Registration: ClinicalTrials.gov NCT04374812) [9]. That study had a waiver for informed consent after assessment via the Health Research Authority integrated research application service (IRAS ref 273185). The ethical review undertaken by the faculty research ethics committee at the University of Southampton (ERGO 52957) for that study was extended for this secondary analysis on the basis that its objectives were aligned with the purpose for which the data had been obtained. The dataset had previously been anonymized with no linkages back to individuals so privacy and confidentiality were respected. The hospital trusts that provided the data gave approvals for this secondary analysis. Dataset construction and all analyses were performed using R (version 4.4.0; R Core Team); further details of the software and packages used can be found in Multimedia Appendix 1.

### Data Sources and Linkage

We analyzed data from adult inpatient wards in 1 hospital trust for admissions from February 2017 to February 2020. The data consisted of anonymized admission records and patient classification assessments made using the SNCT acuity instrument. SNCT acuity assessments had been undertaken in admission units, general wards, and high-care wards but not in intensive care units. The admission units in this hospital were the medical emergency and surgical emergency assessment units, which assess patients before transferring them to a general or high-care ward as required. In the UK, a general ward is an inpatient adult ward not specializing in maternity or psychiatric care. A high-care ward has some high-care beds which are defined as providing level 2 care according to the UK intensive care society, deemed as needing 0.5 WTE of nursing care per bed. A UK intensive care unit, on the other hand, provides the highest level of care, level 3, and this requires one-to-one nursing care. The SNCT acuity assessments data provided counts of patients in a ward for each acuity and dependency category at a point in time. The admission records included each patient’s first NEWS (National Early Warning Score) [10]. NEWS is a patient assessment method in routine use in UK hospitals based on vital signs observations, which nurses use regularly to identify patients at risk of deterioration.

The patient categorization instrument used for ward workload assessments in SNCT involves matching patients to 1 of 5 ordinal categories according to descriptions of acuity and dependency needs. Level 0 is for stable patients with needs met in general wards, level 1a patients are acutely ill patients requiring intervention or those with a greater potential to deteriorate, level 1b patients are stable but are more dependent on nursing care, and level 2 patients are unstable, at risk of deteriorating, and require specialized care. Level 3 patients, who require multiple organ support, did not exist in our dataset of assessments. Each category has a value (called a multiplier) corresponding to a staffing WTE needed to provide 24-hour care after considering annual leave and sickness absence. Multipliers were developed from ward observational studies [11]. One set of multipliers is used for general wards and another for admission units to take into account the extra workload associated with the higher throughput of patients. The multiplier values for the patients in a ward are summed to produce a point-in-time WTE estimate for the ward [4].

All admission records that could be linked by ward and by assessment date and time were included for wards that were routinely carrying out the assessments. Since the assessment records did not record any patient identifiers, linkage to patient records was by ward and time. Linked records were analyzed where the number of patients in the ward assessment matched the number of occupants in the ward according to the patient administration system at the time of the assessment. A comparison of linked and unlinked assessments was conducted to check for bias resulting from this selection process.

### Study Variables

The dependent variable for the prediction model was the WTE calculated by the assessment tool divided by the number of assessed patients. Patients’ data for the assessed patients, as determined by the linkage described earlier, were aggregated to ward level by taking means for continuous variables and proportions for binary variables, as shown in the table. The choice of candidate predictors (Table 1) was made according to data availability, published nurse workload models, and clinical protocols regarding nurse assessments for new ward arrivals. Multi-variable models using all the candidate predictors were built since there was evidence or conceptual reasons for including them, and the objective of the modeling was predictive accuracy rather than quantifying effects of individual predictors. Univariable regressions were performed on the candidate predictors out of interest. The predictors include the use of 2 derived variables: the Charlson comorbidity index [12] and the summary hospital mortality indicator (SHMI) risk [13]. The diagnostic predictors were proportions of patients with a recorded diagnosis belonging to a particular diagnostic group: respiratory conditions, cardiac conditions, renal conditions, cancer, frailty syndromes [14], a self-harm diagnosis, and a history of poisoning. The details of the diagnostic groups are provided in Multimedia Appendix 1. Lastly, an indicator was added for admission units to reflect the alternate set of patient category multipliers used for assessments in them.

**Table 1.** Model predictors.

Category and predictor		Ward aggregation
Demographic		
	Age (mid-point of 5 year age group)	Mean
	Male gender	Proportion
Clinical		
	Admission NEWS^a^	Mean
Pathway		
	Elective admission	Proportion
	Prior length of stay	Median
	Number of ward transfers	Mean
	Number of consultant transfers	Mean
	From admission unit (same day transfer)	Proportion
	From theaters (same day transfer)	Proportion
	From high or intensive care (same day transfer)	Proportion
	New hospital admission (same day, no prior ward)	Proportion
Diagnostic		
	Renal condition	Proportion
	Cardiac condition	Proportion
	Cancer condition	Proportion
	Frailty syndrome: dementia or delirium	Proportion
	Frailty syndrome: dependence or care	Proportion
	Frailty syndrome: incontinence	Proportion
	Frailty syndrome: falls or fractures	Proportion
	Frailty syndrome: pressure ulcers or weight loss	Proportion
	Frailty syndrome: anxiety or depression	Proportion
	Frailty syndrome: mobility problems	Proportion
	Respiratory condition	Proportion
	History of poisoning	Proportion
	Self-harm diagnosis	Proportion
Organizational		
	Admission unit	Binary
Derived		
	SHMI^b^ mortality risk	Mean
	Charlson Comorbidity Index	Mean

a NEWS: National Early Warning Score.

b SHMI: summary hospital mortality indicator.

### Analysis

A generalized additive regression model (GAM) [15] with an identity link function was chosen for the modelling since this could handle nonlinear relationships between the individual predictors and the WTE per patient response variable.

The data were randomly split into training and test sets (75% vs 25%), and a regression model was created on the training set. All the predetermined candidate predictors were used after checking for high pairwise correlations. Removal of any predictor was found to worsen model fit judging by the Akaike information criterion, so all were retained. A calibration was computed to optimize the predictions by means of a smoothing of the model estimates on the assessed ones [16]. The model and the calibration were applied to the test set.

A Bland-Altman plot [17] was constructed and used to look at the agreement between the model prediction and the assessed WTE per patient. When examining this plot by ward subset, 2 wards had more than a third of their predictions outside the limits of agreement. This was a markedly higher proportion of assessments than for the other wards, so these wards were excluded from the datasets, and the GAM model was rebuilt.

## Results

**Figure 1.**
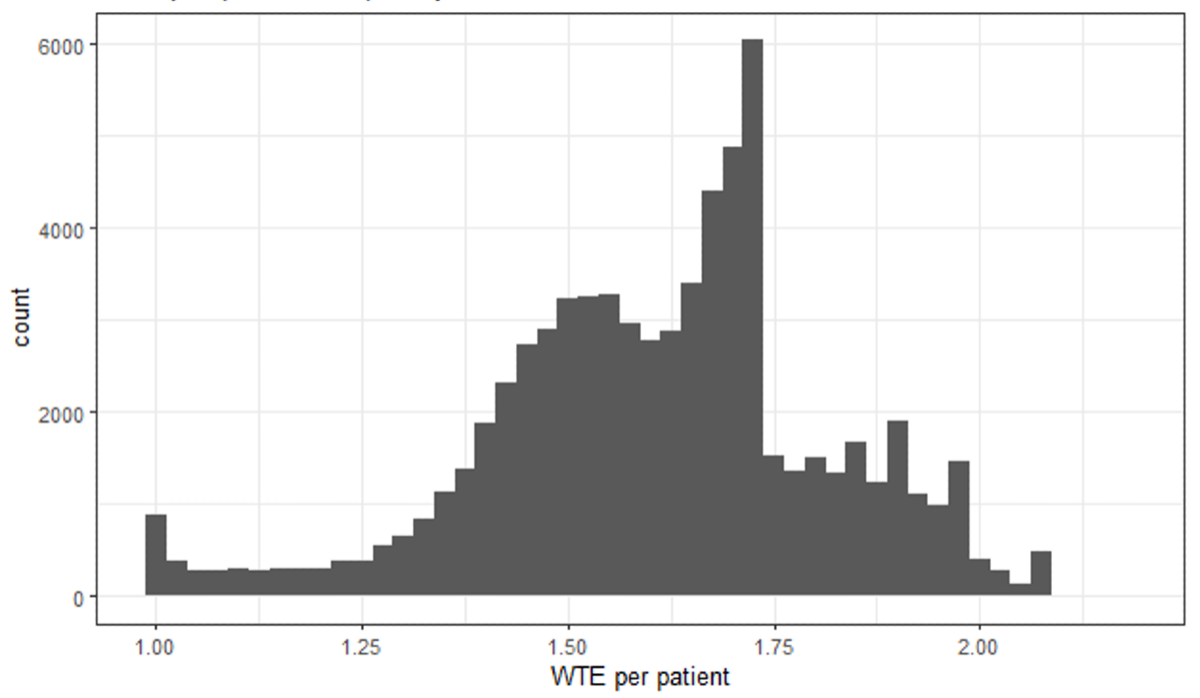
Distribution of whole-time equivalent (WTE) per patient values from safer nursing care tool ward assessments.

The distribution of WTE per patient for all assessments (median 1.63, IQR 1.48-1.72) shows a distinct peak near the value of the multiplier for level 1b (Figure 1).

The data came from 44 wards, including 8 admission units and 4 high-care wards, covering 1,481,801 patient categorizations by care level from 71,027 ward-level assessments relating to 125,595 admissions. Most patient assessments in general wards (n=809,296, 59.3%) were recorded as level 1b (Table 2).

**Table 2.** Percentage of assessed patient categorizations by care level.

	Patient categorizations, n	Level 0, n (%)	Level 1a, n (%)	Level 1b, n (%)	Level 2, n (%)
General or high-care ward	1,364,690	232,620 (17.0)	220,621 (16.2)	809,296 (59.3)	102,153 (7.5)
Admission unit	117,111	29,324 (25.0)	44,505 (38.0)	42,717 (36.5)	565 (0.5)

Requiring the number of ward occupancy records to match the number of patients assessed reduced the dataset by 32% to 48,493 assessments relating to 108,558 admissions; however, the WTE per patient distribution was little changed (median 1.65, IQR 1.51-1.73), suggesting no systematic bias. The ward occupancy and therefore the number of patients per assessment in the matched dataset had a median of 20 with an IQR of 11-29.

A patient might be assessed on a number of occasions in a number of wards during their admission. The cohort of assessed patients consisted of 85.5% (n=92,766) emergency admissions and 46.9% (n=50,877) men, with most coming from the 80‐84 age bracket (Table 3). Long-term conditions were common, with 53.1% (n=57,635) having a Charlson Comorbidity Index of more than 5. The median length of stay was 4.4 (IQR 2.0-10.5) days, and 4.9% (n=5332) died in the hospital.

**Table 3.** Admission descriptives for modeled dataset.

		Value (n=108,558)
Sex (male), n (%)		50,877 (46.9)
Emergency admission, n (%)		92,766 (85.5)
Age group (years), n (%)		
	10‐14	1 (0.0)
	15‐19	1105 (1.0)
	20‐24	2765 (2.5)
	25‐29	2946 (2.7)
	30‐34	3274 (3.0)
	35‐39	3275 (3.0)
	40‐44	3212 (3.0)
	45‐49	4525 (4.2)
	50‐54	5775 (5.3)
	55‐59	7031 (6.5)
	60‐64	7405 (6.8)
	65‐69	8663 (8.0)
	70‐74	11,997 (11.1)
	75‐79	11,830 (10.9)
	80‐84	12,886 (11.9)
	85‐89	11,973 (11.0)
	90‐120	9895 (9.1)
Charlson Index, n (%)		
	0	32,933 (30.3)
	1-5	17,734 (16.3)
	>5	57,635 (53.1)
	Missing	256 (0.2)
SHMI risk^a^ (%), mean (SD)		7.0 (10.0)
NEWS2^b^, n (%)		
	Zero, 0	60,179 (55.4)
	Low, 1‐4	36,789 (33.9)
	Med, 5‐6	6708 (6.2)
	High, 7‐20	3343 (3.1)
	(Missing)	1539 (1.4)
Length of stay (days), median (IQR)		4.4 (2.0-10.5)
Hospital death, n (%)		5332 (4.9)

a Summary Hospital Mortality Indicator.

b National Early Warning Score (version 2).

**Figure 2.**
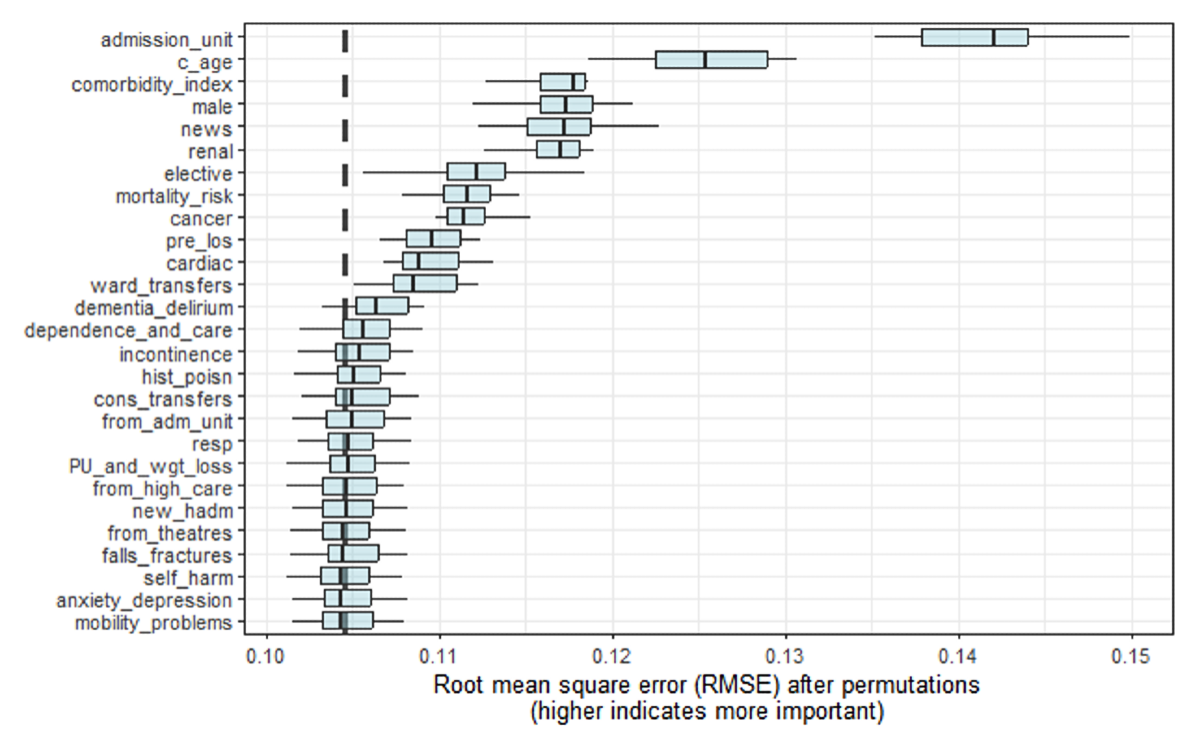
Relative contribution each predictor makes to the model fit.

We fitted a GAM to the training set to model the nursing workload measured in WTE per patient associated with the set of patients in each manual ward assessment. When applied to the test set of 11,592 ward assessments from 42 wards and a calibration applied, the mean absolute error of the predictions was 0.078 WTE per patient (a mean absolute percentage error of 4.9% with a 95th centile of 13.9%). The root mean square error was 0.106 WTE per patient, and the percentage of variance explained by the model was 65%. The predictors that contributed most to explaining the workload (Figure 2) were the admission unit indicator, mean patient age, mean Charlson comorbidity index, the proportion of men, the mean NEWS, and the proportion of patients with a diagnosed renal condition. The relative contribution of each predictor was computed by calculating the root mean square error if removed from the model.

Univariable regressions of each of the predictors showed them all to be significant, apart from transfer from an admission unit on the day of assessment (Table S4 in Multimedia Appendix 1). All predictors were retained in the GAM model.

**Figure 3.**
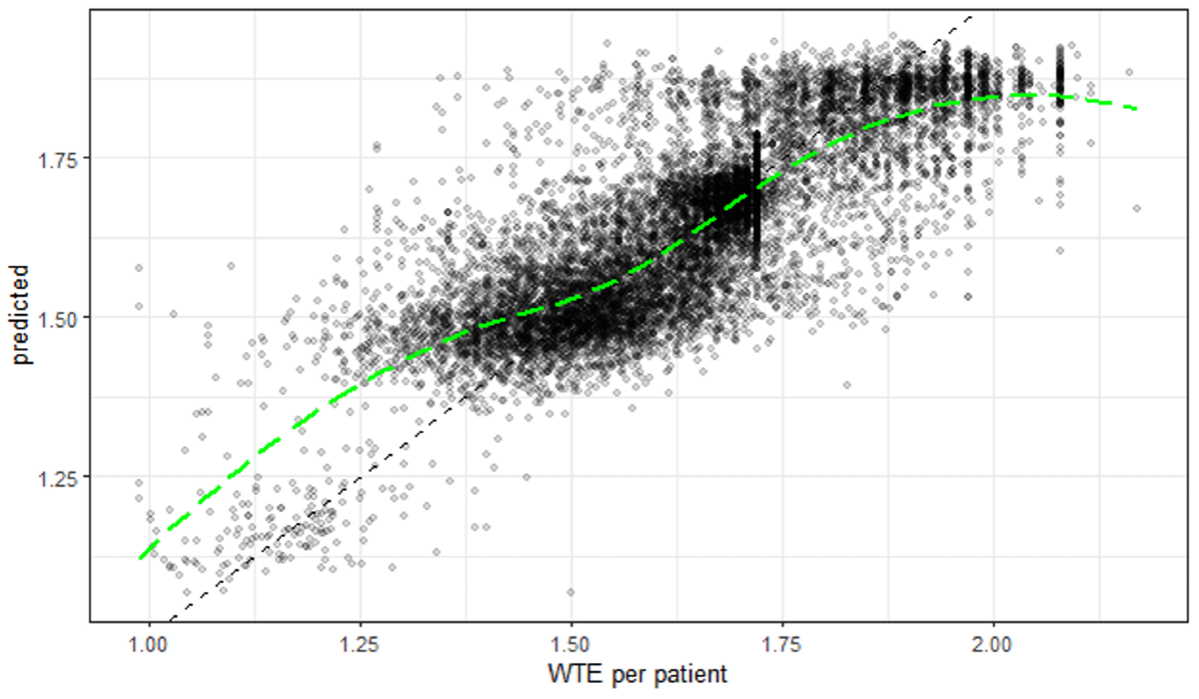
Calibration of predictions in the test set. WTE: whole-time equivalent.

A calibration plot of predictions in the test set against the recorded WTE per patient with the addition of a loess smoothing line forming a calibration curve [18] shows that there was some underestimation of workload at high values (Figure 3).

**Figure 4.**
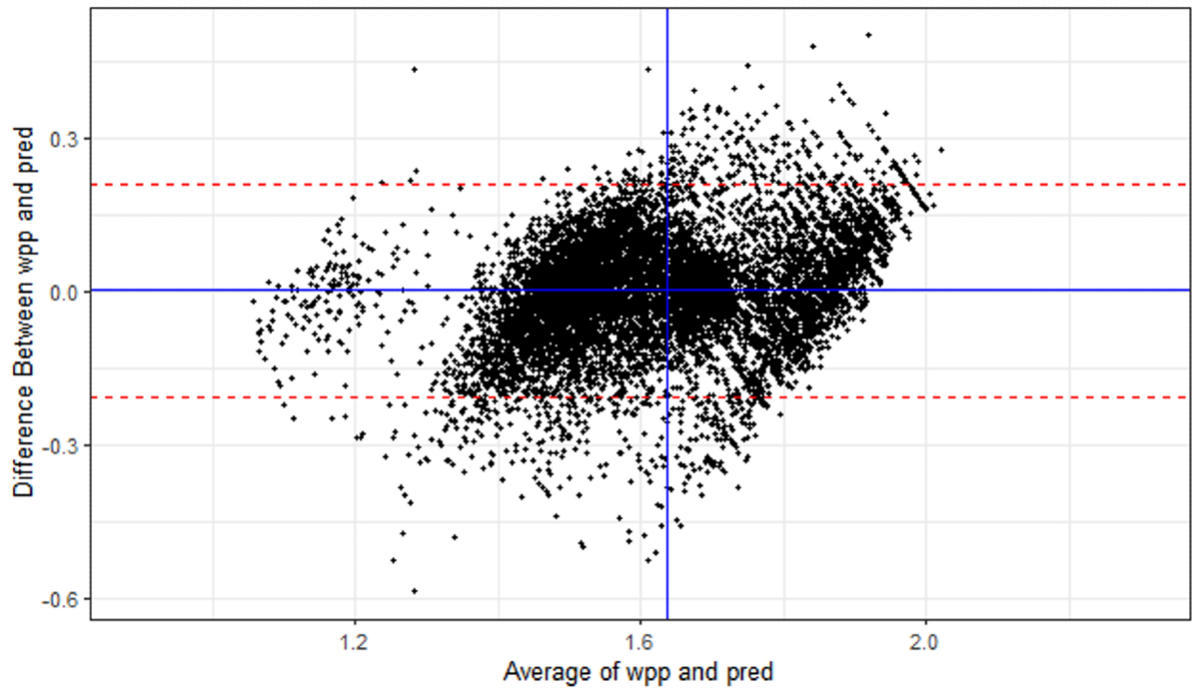
Bland-Altman plot of test model assessments and predictions. pred: model prediction; wpp: whole-time equivalent per patient.

A Bland-Altman plot of differences between the recorded WTE per patient and model predictions in the test set (Figure 4) had a mean difference of 0.002 WTE per patient. The limits of agreement are at ±0.21 WTE per patient, meaning 95% of assessments fall in this range.

## Discussion

### Main Findings

In this study, we used a limited set of routine patient data to estimate the demand for nursing staff on general and high-care hospital wards and admission units. We found that data on patient demographics, routes of admission, ward transfers, diagnoses, and admission NEWS, which are available in hospital systems, could be used to estimate the results of assessments made using a PCS with a moderate degree of precision. The predictions of WTE per patient were on average within 5% of the recorded estimates when applied to a test set. Calibration “in the large” was good in the test set, with the mean prediction being only 0.002 WTE per patient less than the recorded mean [18]. However, a calibration curve showed that the model tended to underestimate at higher workloads. This would be the case if some dimensions of patients’ care needs in more acute wards were not being adequately represented in the dataset. A “by ward” sub-analysis of the model estimates showed that the surgical high care unit and the renal transplant unit were the 2 wards where the model underestimated the most (as proportions of predictions). This might indicate the model is lacking variables that adequately capture the acuity and dependency of patients after major surgery. If the model were to be used as it is, a professional judgment framework, such as that published by Saville et al [19], should be used, which might recommend considering adjustment of the estimates where they exceeded 1.75 WTE or where there were high-care beds in the ward.

The predictors were not chosen to be orthogonal; they clearly have correlations when considering their potential effect on nurse workload. That means Figure 2 explaining their contribution to estimating workload needs to be interpreted with care. It is not surprising that the admission unit indicator makes the largest contribution since an approximately 10% uplift in nurse staffing for admission units is baked into the PCS, which determines the target WTE per patient. It is perhaps expected that the age, comorbidity index, and admission NEWS case mix variables of ward occupancy influence estimates of workload. However, the marked contribution of gender invites comment. It may reflect staffing in single-sex wards dealing with gynecological or urological conditions.

While overall fit was good, the Bland-Altman plot shown in Figure 4 revealed a distinct artifact of 45-degree downward-sloping lines, which arises from ranges of continuous prediction values estimating a given workload derived from the discrete set of multiplier values in the PCS and the numbers of patients in wards. There is a degree of subjectivity in categorizing patients in the PCS into one of the 4 levels, and there is a possibility that there was some up-coding of assessments in which patients were put into a higher category, for example, level 1b rather than level zero, which might boost a ward’s chances of getting more resources [20]. The high proportion of patient assessments in the level 1b category shown in Table 2 possibly suggests some of these were erroneous, although patients with dependent care needs may have longer than average stays with opportunity for more categorizations at that level.

In conversations, safe staffing managers and leads at a number of hospitals have expressed concerns regarding the accuracy and reliability of manual patient categorizations by ward staff. An automated workload prediction system would be objective and eliminate or reduce a source of bias. It would also remove the need for training staff in carrying out the assessments and retraining them when assessment methods are revised.

The OPCq and Perroca patient classification instruments for assessing patient care needs are more structured than the SNCT instrument, as they require scoring a patient across several domains before determining an overall category. The drawback to this rigor is that the assessments take longer. One nurse quoted in the publication by Ayan et al (2024) [21] concerning the Perroca instrument said: “Since we have been using it continuously, it does not take me much time now. It only takes five minutes to assess a patient; it couldn’t be easier.”

Our observational data (unpublished) from another study [22] suggests that assessing all patients on a 30-bedded ward 3 times per day with SNCT categorizations could occupy a senior nurse for up to 30 minutes per day, plus extra time for data entry.

The identification of 2 wards with outlier predictions is of interest. In the private patient unit, the model tended to predict higher staffing requirements than those identified in the assessments. This could arise because of a selection effect, whereby the occurrence of more elective procedures on more medically fit patients was not reflected in the variables used for this population. In contrast, the predicted workload estimates for the surgical high care unit tended to be too low, and this might mean the model is not adjusting adequately for these acutely ill patients. Additional predictors to adjust for operational severity might improve the predictions.

The surprising accuracy of the predictions on limited data opens up the possibility of automated near-time assessments and consequently freeing up some nursing time. The feasibility of implementing the prediction algorithm in real-time or near-time is dependent on the data items being recorded and accessible in a timely manner. In the United Kingdom, basic patient admission data is usually recorded in near-time in a patient administration system with electronic interfacing, though the clinical coding of diagnoses in the same system is often much later. However, the diagnoses the algorithm uses are largely for long-term conditions or conditions known on presentation, in which case they may already be present in the electronic clinical history or recorded in nursing or clinical handover documentation. A National Health Service (NHS) England-sponsored digital maturity assessment of UK hospital trusts reports that 32 of 132 acute trusts have electronic nursing and physician documentation [23], and that number is rising, so real-time electronic provision of patient conditions is becoming more widespread.

### Comparison With Prior Work

The approach taken in this study differs from previous studies that built models or made estimates of actual historic staffing levels [24-26]. In contrast, our approach was to build a model of estimated staffing requirements trained on the records of an existing staffing estimation tool [27]. Unlike the classifier model built by Rosa [7], which was trained on values from the PPCI to make patient categorizations, ours was a prediction model of ward staffing requirements. While the prediction of PPCI values was only moderately accurate (72% correct classification), in our study, the magnitude of errors for estimating ward staffing requirements was, on average, small, with a mean average percentage error of less than 5% and 95% of estimates within 14% of the assessed value. The required precision is an open question, but staffing within ±15% of measured demand is used as a criterion to define acceptable variation in one widely used system [28].

The method we have used to build a model that is trained on the ward workload is flexible and would work for other patient acuity and dependency scoring instruments that assign an ordinal value for the patient’s care needs.

### Limitations

The dataset was limited in terms of longitudinal clinical data regarding patient conditions. While it contained the NEWS on admission, it did not have subsequent scores or individual vital signs. Without time-varying variables such as NEWS and other assessments that reflect a patient’s evolving acuity, the model is relying on the ward case mix not varying from the average in terms of proportions of patients medically fit for discharge or proportions deteriorating. These 2 cases work in opposite directions in terms of workload, those patients who are medically fit probably requiring less nursing care, while those who deteriorate probably requiring more nursing care. A systematic bias in the model performance is not easy to determine without further information. Also, as noted earlier in the main findings, the poor performance of the model in the private patient unit and in surgical high care might result from the model lacking information on surgical procedures and operation severity. This limits the settings in which the current model might be used.

Another limitation that might have impacted the accuracy of the predictions was the reliability of the recorded patient classifications. The use of the acuity categorization tool for estimating staffing requirements in the dataset was new to the hospital at the time; hence, the reliability of the assessment may have depended for a period on the effectiveness of training given to ward assessors and monitoring of the use of the tool.

The robustness and generalizability of the model need to be checked by assessing its performance using data from other hospitals. It is possible that variation in patient case mix and in how hospitals apply SNCT patient acuity categorizations could affect model performance. It should also be noted that while the aim of this study was to estimate workload that matched the existing SNCT-based assessments, the tool in use did not refine workload requirements by skill mix, for example, advising how many registered nurses and how many nurse support staff might meet the requirements, nor about requirements for specialist skills. This would be a useful step in matching requirements to available resources and might be the subject of further work.

### Conclusions

This research demonstrated a way of building a moderately accurate prediction model of nurse staffing requirements from a limited dataset of routine data by training the model on records of an established patient acuity and dependency patient classification tool. The results demonstrate the potential for automating assessments of nurse staffing requirements from routine data, reducing time spent on this nonclinical overhead, and improving monitoring of real-time staffing pressures.

## Supplementary material

10.2196/71666 Multimedia Appendix 1 Diagnosis groupings, results, and software.

## Abbreviations

GAM: generalized additive regression model
NEWS: National Early Warning Score
OPCq: Oulu Patient Classification qualisan
PCS: patient classification system
PPCI: Perroca Patient Classification Instrument
SHMI: summary hospital mortality indicator
SNCT: safer nursing care tool
WTE: whole-time equivalent
