# Supplementary material for: Estimating Nurse Workload Using a Predictive Model From Routine Hospital Data: Algorithm Development and Validation
Source: JMIR Med Inform. 2025 Jul 31;13:e71666. doi: 10.2196/71666 (PMC12314723; doi:10.2196/71666)
Supplement: Multimedia Appendix 1 [file medinform-v13-e71666-s001.docx]

## Multimedia Appendix 1

## Diagnosis Groupings

### Frailty Syndromes

The 7 Frailty Syndromes were defined in the paper by Soong [14]: Dementia and Delirium, Mobility Problems, Falls and Fractures, Pressure Ulcers and Weight Loss, Incontinence, Dependence and Care, Anxiety and Depression

### Respiratory conditions

ICD-10 Codes: J41-J44, J60-J68

### History of Poisoning or Self-harm

ICD-10 Codes: Z915

### Self-harm

ICD-10 Codes: X60-X69, X70-X79, X80-X84

### Renal, Cancer and Cardiac conditions

These were defined using the SHMI diagnostic groups [13] which were in turn defined by the clinical condition groups specified by the Agency for Healthcare Research and Quality [29]

#### Renal

| SHMI grp | CCS Conditions |  |
| --- | --- | --- |
| 99 | 157 | Acute and unspecified renal failure |
| 100 | 156, 158 | Nephritis; nephrosis; renal sclerosis, Chronic renal failure |
| 102 | 160, 161, 162 | Diseases of kidneys and ureters, bladder and urethra |

#### Cancer

| SHMI grp | CCS Conditions |  |
| --- | --- | --- |
| 7 | 11 | Cancer of head and neck |
| 8 | 12 | Cancer of oesophagus |
| 9 | 13 | Cancer of stomach |
| 10 | 14 | Cancer of colon |
| 11 | 15 | Cancer of rectum and anus |
| 12 | 16 | Cancer of liver and intrahepatic bile duct |
| 13 | 17 | Cancer of pancreas |
| 14 | 18 | Cancer of other GI organs; peritoneum |
| 15 | 19 | Cancer of bronchus; lung |
| 16 | 20 | Cancer; other respiratory and intrathoracic |
| 17 | 22, 23 | Melanomas, other cancer of skin |
| 18 | 24 | Cancer of breast |
| 19 | 25 | Cancer of uterus |
| 20 | 26, 28 | Cancer of female genital organs |
| 21 | 27 | Cancer of ovary |
| 22 | 29, 30, 31 | Cancer of male reproductive organs |
| 23 | 32 | Cancer of bladder |
| 24 | 33, 34 | Cancer of urinary organs |
| 25 | 35 | Cancer of brain and nervous system |
| 26 | 37 | Hodgkin's disease |
| 27 | 39 | Leukemias |
| 28 | 40 | Multiple myeloma |
| 29 | 41, 45 | Other cancer (primary) |
| 30 | 42 | Secondary malignancies |
| 31 | 21, 36, 43 | Cancer of bone, thyroid and malignant neoplasm |
| 32 | 44, 167 | Neoplasms (unspecified), Nonmalignant breast conditions |
| 33 | 46, 47 | Benign neoplasm |

#### Cardiac

| SHMI grp | CCS Conditions |  |
| --- | --- | --- |
| 57 | 100 | Acute myocardial infarction |
| 58 | 101 | Coronary atherosclerosis and other heart disease |
| 59 | 102 | Nonspecific chest pain |
| 60 | 103 | Pulmonary heart disease |
| 61 | 104 | Other and ill-defined heart disease |
| 62 | 105 | Conduction disorders |
| 63 | 106 | Cardiac dysrhythmias |
| 64 | 107 | Cardiac arrest and ventricular fibrillation |
| 65 | 108 | Congestive heart failure; nonhypertensive |

## Appendix Results

Table 4: Results of univariable regressions

|  | **Predictor** | **Coeff** | **95% Conf. Int** | **Sign.** |
| --- | --- | --- | --- | --- |
| **Demographics** | Age (md-point of 5 yr age group) | 0.001 | (0.001 to 0.002) | p<0.001 |
|  | male | 0.03 | (0.03 to 0.04) | p<0.001 |
| **Clinical** | NEWS | 0.04 | (0.03 to 0.04) | p<0.001 |
| **Pathway** | Elective (scheduled) admission | -0.33 | (-0.34 to -0.32) | p<0.001 |
|  | prior length of stay | 0.001 | (0.001 to 0.002) | p<0.001 |
|  | Prior no. of ward_transfers | -0.07 | (-0.07 to -0.06) | p<0.001 |
|  | Prior no. of consultant_transfers | -0.04 | (-0.04 to -0.03) | p<0.001 |
|  | from_high_care (that day) | -1.04 | (-1.14 to -0.94) | p<0.001 |
|  | from_theatres (ditto) | -1.22 | (-1.38 to -1.06) | p<0.001 |
|  | from_adm_unit (ditto) | -0.02 | (-0.06 to 0.02) | p=0.260 |
|  | new_hospital_admission (ditto) | 0.35 | (0.34 to 0.37) | p<0.001 |
| **Diagnostic** | renal | 0.48 | (0.45 to 0.50) | p<0.001 |
|  | cancer | -0.05 | (-0.06 to -0.04) | p<0.001 |
|  | cardiac | -0.12 | (-0.13 to -0.11) | p<0.001 |
|  | respiratory | 0.23 | (0.21 to 0.24) | p<0.001 |
|  | self_harm | 0.72 | (0.64 to 0.80) | p<0.001 |
|  | history_of_poisoning | 0.48 | (0.41 to 0.54) | p<0.001 |
|  | dementia, delirium | 0.12 | (0.11 to 0.12) | p<0.001 |
|  | mobility_problems | 0.15 | (0.13 to 0.16) | p<0.001 |
|  | falls, fractures | 0.07 | (0.06 to 0.08) | p<0.001 |
|  | Pressure_Ulcers, weight_loss | 0.21 | (0.19 to 0.24) | p<0.001 |
|  | incontinence | 0.31 | (0.29 to 0.32) | p<0.001 |
|  | dependence_and_care | -0.10 | (-0.11 to -0.08) | p<0.001 |
|  | anxiety, depression | 0.03 | (0.01 to 0.05) | p=0.003 |
| **Organisational** | admission_unit | 0.19 | (0.18 to 0.19) | p<0.001 |
| **Derived** | comorbidity_index | 0.01 | (0.01 to 0.01) | p<0.001 |
|  | mortality_risk | 0.86 | (0.82 to 0.90) | p<0.001 |

## Appendix Software

All analyses were performed using R Statistical Software (v4.4.0)^1^ in the RStudio integrated development environment (v2024.12.0.467)^2^. The tidyverse R package (v2.0.0)^3^ was used for data wrangling, mgcv (v1.9-1)^4^ for GAM regression and yardstick (v1.31)^5^ for prediction metrics. Descriptive statistics used the finalfit R package (v1.0.8)^6^. The rmarkdown R package (v2.28)^7^ was used to format output in the preparation of this paper.

1 R Core Team (2024). _R: A Language and Environment for Statistical Computing_. R Foundation for Statistical Computing, Vienna, Austria. <https://www.R-project.org/>.

2 Posit team (2024). RStudio: Integrated Development Environment for R. Posit Software, PBC, Boston, MA. URL http://www.posit.co/.

3 Wickham H, Averick M, Bryan J, Chang W, McGowan LD, François R, Grolemund G, Hayes A, Henry L, Hester J, Kuhn M, Pedersen TL, Miller E, Bache SM, Müller K, Ooms J, Robinson D, Seidel DP, Spinu V, Takahashi K, Vaughan D, Wilke C, Woo K, Yutani H (2019). “Welcome to the tidyverse.” _Journal of Open Source Software_, *4*(43), 1686. doi:10.21105/joss.01686 https://doi.org/10.21105/joss.01686

4 Wood, S.N. (2017) Generalized Additive Models: An Introduction with R (2nd edition). Chapman and Hall/CRC.

5 Kuhn M, Vaughan D, Hvitfeldt E (2024). _yardstick: Tidy Characterizations of Model Performance_. R package version 1.3.1, <https://CRAN.R-project.org/package=yardstick>.

6 Harrison E, Drake T, Pius R (2023). _finalfit: Quickly Create Elegant Regression Results Tables and Plots when Modelling_. R package version 1.0.7, <https://CRAN.R-project.org/package=finalfit>.

7 Allaire J, Xie Y, Dervieux C, McPherson J, Luraschi J, Ushey K, Atkins A, Wickham H, Cheng J, Chang W, Iannone R (2023). _rmarkdown: Dynamic Documents for R_. R package version 2.25, <https://github.com/rstudio/rmarkdown>.
